# Redetermination of the crystal structure of caesium tetra­fluorido­bromate(III) from single-crystal X-ray diffraction data

**DOI:** 10.1107/S2414314620001145

**Published:** 2020-01-31

**Authors:** Artem V. Malin, Sergei I. Ivlev, Roman V. Ostvald, Florian Kraus

**Affiliations:** a National Research Tomsk Polytechnic University, 30 Lenina avenue, 634050 Tomsk, Russian Federation; b Fachbereich Chemie, Philipps-Universität Marburg, Hans-Meerwein-Strasse 4, 35032 Marburg, Germany; Vienna University of Technology, Austria

**Keywords:** crystal structure, caesium, tetra­fluorido­bromate(III), redetermination

## Abstract

Caesium tetra­fluorido­bromate(III), CsBrF_4_, was crystallized in form of small blocks by melting and recrystallization. The crystal structure of CsBrF_4_ was redetermined from single-crystal X-ray diffraction data. In comparison with a previous study based on powder X-ray diffraction data [Ivlev *et al.* (2013 ▸). *Z. Anorg. Allg. Chem*. **639**, 2846–2850], bond lengths and angles were determined with higher precision, and all atoms were refined with anisotropic displacement parameters. It was confirmed that the structure of CsBrF_4_ contains two square-planar [BrF_4_]^−^ anions each with point group symmetry *mmm*, and a caesium cation (site symmetry *mm*2) that is coordinated by twelve fluorine atoms, forming an anti­cubocta­hedron. CsBrF_4_ is isotypic with CsAuF_4_.

## Abstract 2

The crystal structure of CsBrF_4_ was determined from single-crystal X-ray diffraction data collected at 100 K and is compared with previous models based on powder X-ray diffraction data.

## Graphical abstract

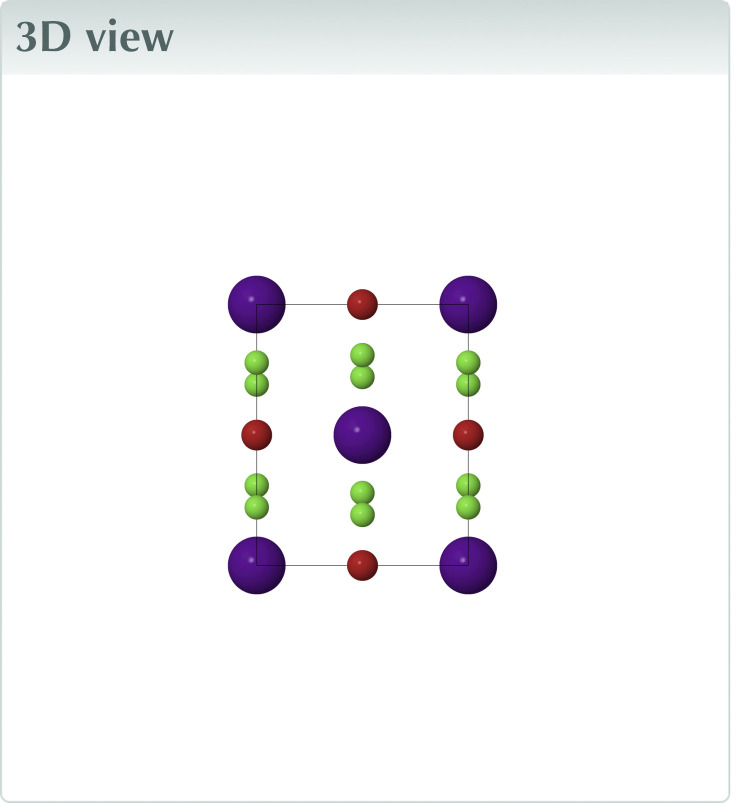

## Structure description

The first report of unit-cell parameters of CsBrF_4_ from powder X-ray diffraction data was given by Popov *et al.* (1987 ▸). They indexed the powder pattern using a primitive tetra­gonal unit cell with lattice parameters of *a* = 9.828 (3), *c* = 7.166 (5) Å, *V* = 692.2 (3) Å^3^ (temperature not given). These lattice parameters are quite different compared to those of other known alkali metal tetra­fluorido­bromates(III) that crystallize in the KBrF_4_ structure type [KBrF_4_, *I*4/*mcm* (No. 140), *a* = 6.174 (2), *c* = 11.103 (2) Å, *V* = 423 Å^3^; Siegel, 1956 ▸], and consequently CsBrF_4_ is not isotypic with KBrF_4_ on basis of the data provided by Popov *et al.* (1987 ▸). However, neither the crystal structure nor other crystallographic details of CsBrF_4_ were given at that time.

Recently, we have determined the crystal structure of CsBrF_4_ from powder X-ray diffraction (PXRD) data where we could only refine the F atoms isotropically (Ivlev *et al.*, 2013 ▸). We have shown that CsBrF_4_ is isotypic with CsAuF_4_ (Schmidt & Müller, 2004 ▸) and crystallizes in the space group *Immm* (No. 71) with lattice parameters *a* = 5.6413 (8), *b* = 6.8312 (9), *c* = 12.2687 (17) Å, *V* = 472.79 (11) Å^3^, *Z* = 4 at 293 K. These lattice parameters are not related to the unit cell reported by Popov *et al.* (1987 ▸). We assume that their powder pattern probably contained impurity lines, *e.g*. from possible hydrolysis products, which led to erroneous indexing. Here we present the results of a redetermination of the crystal structure of CsBrF_4_ from single-crystal X-ray diffraction data at 100 K, leading to bond lengths and angles with higher precision, and with all atoms refined with anisotropic displacement parameters.

**Figure 1:**
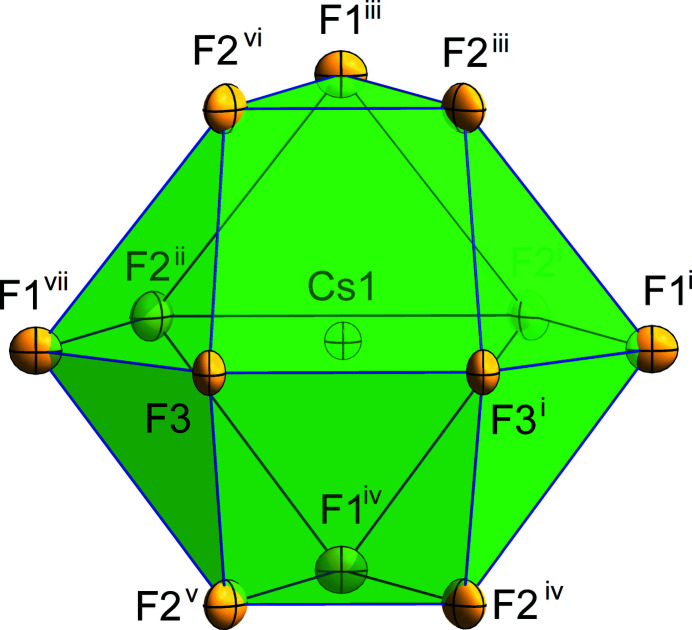
The anti­cubocta­hedron around the caesium cation. Displacement ellipsoids are shown at the 70% probability level. [Symmetry codes: (i) −*x* + 1, −*y* + 1, −*z* + 1; (ii) *x*, *y*, −*z* + 1; (iii) *x* + 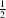 , *y* + 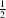 , *z* + 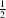 ; (iv) *x * − 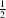 , *y* + 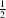 , *z* + 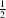 ; (v) −*x* + 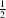 , −*y* + 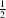 , *z* + 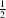 ; (vi) −*x* + 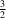 , −*y* + 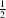 , *z* + 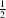 ; (vii) −*x* + 1, −*y*, −*z* + 1.]

The unit-cell parameters of CsBrF_4_ obtained from single-crystal X-ray diffraction data (Table 1 ▸) are expectedly smaller than those from the PXRD data at 293 K. The crystal structure contains two different square-planar [BrF_4_]^−^ anions, the planes of which are parallel and rotated by about 45° with respect to each other. The first anion consists of one bromine(III) atom (Br1) on the special 2*d* (*mmm*) Wyckoff position and two fluorine atoms F1 and F3 on the special 4*j* (*mm*2) and 4*g* (*m*2*m*) Wyckoff positions, respectively. As a result of symmetry restrictions, the F—Br—F angle is exactly 90°. The Br1—F bond lengths are 1.8852 (13) and 1.9020 (15) Å [*cf*. 1.94 (4) and 1.97 (4) Å from PXRD data]. The second [BrF_4_]^−^ anion contains one bromine(III) atom (Br2) on the special 2*b* (*mmm*) Wyckoff position and one fluorine atom (F2) on the special 8*l* (*m*..) Wyckoff position. The anion is slightly distorted in-plane, resulting in an almost rectangular structure with F2—Br2—F2 angles of 87.96 (7) and 92.04 (7)° and a Br2—F2 bond length of 1.8907 (10) Å [*cf*. 87.6 (13) and 92.4 (13)°, 1.96 (3) Å from PXRD data]. In general, the bond lengths and angles of the [BrF_4_]^−^ anions in CsBrF_4_ are in good correspondence with other known tetra­fluorido­bromates(III) [see Table 2 in Ivlev & Kraus (2018 ▸), and references therein]. The caesium cation occupies the special 4*i* (*mm*2) Wyckoff position and is coordinated by twelve fluorine atoms. The resulting coordination polyhedron is an anti­cubocta­hedron (Fig. 1 ▸). The Cs⋯F distances are in the range 2.9615 (11) to 3.4784 (4) Å [*cf*. 3.011 (1) to 3.605 (1) from PXRD data].

**Figure 2:**
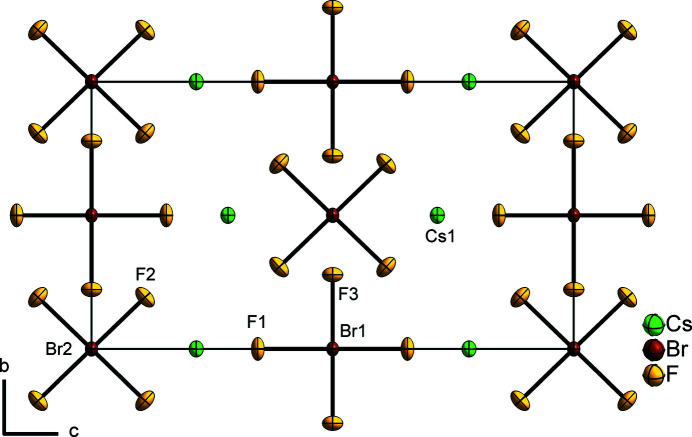
The crystal structure of CsBrF_4_ in a projection along the *a* axis. Displacement ellipsoids are shown at the 70% probability level.

The crystal structure of CsBrF_4_ is shown in Fig. 2 ▸.

## Synthesis and crystallization

Caesium tetra­fluorido­bromate(III) was synthesized by direct reaction of bromine trifluoride with caesium chloride. The reaction was carried out under Freon-113, which acted as a protective layer against hydrolysis and as a heat absorber. The mixture of CsCl and BrF_3_ was kept in a closed Teflon vessel. After three days the Freon was removed by vacuum distillation and CsBrF_4_ was obtained as a solid white residue. The powder was melted at 483 K and cooled down to room temperature. Single crystals of CsBrF_4_ were obtained as small blocks after crushing the solid lumps.

## Refinement

Details of data collection and structure refinement are given in Table 1 ▸. Coordinates and atom labelling were taken from the previous refinement from PXRD data (Ivlev *et al.*, 2013 ▸).

## Supplementary Material

Crystal structure: contains datablock(s) I. DOI: 10.1107/S2414314620001145/wm4121sup1.cif

Structure factors: contains datablock(s) I. DOI: 10.1107/S2414314620001145/wm4121Isup2.hkl

CCDC reference: 1980292

Additional supporting information:  crystallographic information; 3D view; checkCIF report

## Figures and Tables

**Table 1:** Experimental details

Crystal data	
Chemical formula	CsBrF_4_
*M*_r_	288.82
Crystal system, space group	Orthorhombic, *I* *m* *m* *m*
Temperature (K)	100
*a*, *b*, *c* (Å)	5.5075 (3), 6.7890 (3), 12.2572 (6)
*V* (Å^3^)	458.30 (4)
*Z*	4
Radiation type	Mo *K*α
μ (mm^−1^)	16.75
Crystal size (mm)	0.11 × 0.09 × 0.06

Data collection	
Diffractometer	Bruker D8 QUEST
Absorption correction	Multi-scan (*SADABS*; Krause *et al.*, 2015 ▸)
*T*_min_, *T*_max_	0.330, 0.558
No. of measured, independent and observed [*I* > 2σ(*I*)] reflections	8570, 669, 622
*R*_int_	0.029
(sin θ/λ)_max_ (Å^−1^)	0.835

Refinement	
*R*[*F* ^2^ > 2σ(*F* ^2^)], *wR*(*F* ^2^), *S*	0.012, 0.022, 1.12
No. of reflections	669
No. of parameters	26
Δρ_max_, Δρ_min_ (e Å^−3^)	1.20, −0.88

Computer programs: *APEX3* and *SAINT* (Bruker, 2018 ▸), *SHELXL* (Sheldrick, 2015 ▸), *DIAMOND* (Brandenburg, 2019 ▸) and *publCIF* (Westrip, 2010 ▸); coordinates taken from previous refinement.

## full crystallographic data

### Crystal data

**Table:**

CsBrF_4_	*D*_x_ = 4.186 Mg m^−^^3^
*M**_r_* = 288.82	Mo *K*α radiation, λ = 0.71073 Å
Orthorhombic, *I**m**m**m*	Cell parameters from 2913 reflections
*a* = 5.5075 (3) Å	θ = 3.3–36.8°
*b* = 6.7890 (3) Å	µ = 16.75 mm^−^^1^
*c* = 12.2572 (6) Å	*T* = 100 K
*V* = 458.30 (4) Å^3^	Block, colorless
*Z* = 4	0.11 × 0.09 × 0.06 mm
*F*(000) = 504	

### Data collection

**Table:**

Bruker D8 QUEST diffractometer	622 reflections with *I* > 2σ(*I*)
Radiation source: microfocus X-ray tube	*R*_int_ = 0.029
ω and φ scans	θ_max_ = 36.4°, θ_min_ = 3.3°
Absorption correction: multi-scan (*SADABS*; Krause *et al.*, 2015)	*h* = −9→9
*T*_min_ = 0.330, *T*_max_ = 0.558	*k* = −11→11
8570 measured reflections	*l* = −20→20
669 independent reflections	

### Refinement

**Table:**

Refinement on *F*^2^	Primary atom site location: structure-invariant direct methods
Least-squares matrix: full	*w* = 1/[σ^2^(*F*_o_^2^) + (0.0065*P*)^2^ + 0.5686*P*] where *P* = (*F*_o_^2^ + 2*F*_c_^2^)/3
*R*[*F*^2^ > 2σ(*F*^2^)] = 0.012	(Δ/σ)_max_ = 0.001
*wR*(*F*^2^) = 0.022	Δρ_max_ = 1.20 e Å^−^^3^
*S* = 1.12	Δρ_min_ = −0.88 e Å^−^^3^
669 reflections	Extinction correction: SHELXL-2018/3 (Sheldrick 2015), Fc^*^=kFc[1+0.001xFc^2^λ^3^/sin(2θ)]^-1/4^
26 parameters	Extinction coefficient: 0.00232 (16)
0 restraints	

### Special details

**Table:**

Geometry. All esds (except the esd in the dihedral angle between two l.s. planes) are estimated using the full covariance matrix. The cell esds are taken into account individually in the estimation of esds in distances, angles and torsion angles; correlations between esds in cell parameters are only used when they are defined by crystal symmetry. An approximate (isotropic) treatment of cell esds is used for estimating esds involving l.s. planes.

### Fractional atomic coordinates and isotropic or equivalent isotropic displacement parameters (Å^2^)

**Table:**

	*x*	*y*	*z*	*U*_iso_*/*U*_eq_	
Cs1	0.500000	0.500000	0.71714 (2)	0.01008 (4)	
Br1	0.500000	0.000000	0.500000	0.00720 (6)	
Br2	0.500000	0.000000	0.000000	0.00760 (6)	
F1	0.500000	0.000000	0.34482 (12)	0.0155 (3)	
F2	0.500000	0.19339 (16)	0.11100 (9)	0.0169 (2)	
F3	0.500000	0.2777 (2)	0.500000	0.0141 (3)	

### Atomic displacement parameters (Å^2^)

**Table:**

	*U*^11^	*U*^22^	*U*^33^	*U*^12^	*U*^13^	*U*^23^
Cs1	0.01130 (6)	0.01075 (6)	0.00821 (7)	0.000	0.000	0.000
Br1	0.00756 (12)	0.00827 (11)	0.00578 (13)	0.000	0.000	0.000
Br2	0.00816 (12)	0.00841 (11)	0.00624 (13)	0.000	0.000	0.000
F1	0.0179 (7)	0.0216 (7)	0.0069 (6)	0.000	0.000	0.000
F2	0.0201 (5)	0.0160 (4)	0.0146 (5)	0.000	0.000	−0.0073 (4)
F3	0.0164 (6)	0.0087 (6)	0.0172 (7)	0.000	0.000	0.000

### Geometric parameters (Å, º)

**Table:**

Cs1—F2^i^	2.9615 (11)	Cs1—F1^vii^	3.4784 (4)
Cs1—F2^ii^	2.9615 (11)	Cs1—F1^i^	3.4784 (4)
Cs1—F3^i^	3.0597 (7)	Br1—F3	1.8852 (13)
Cs1—F3	3.0597 (7)	Br1—F3^vii^	1.8853 (13)
Cs1—F1^iii^	3.1674 (8)	Br1—F1^vii^	1.9020 (15)
Cs1—F1^iv^	3.1674 (8)	Br1—F1	1.9020 (15)
Cs1—F2^v^	3.3166 (7)	Br2—F2^viii^	1.8907 (10)
Cs1—F2^iii^	3.3166 (7)	Br2—F2^ix^	1.8907 (10)
Cs1—F2^iv^	3.3166 (7)	Br2—F2^x^	1.8907 (10)
Cs1—F2^vi^	3.3166 (7)	Br2—F2	1.8907 (10)
			
F2^i^—Cs1—F2^ii^	89.32 (4)	F1^iv^—Cs1—F1^i^	96.194 (16)
F2^i^—Cs1—F3^i^	105.79 (3)	F2^v^—Cs1—F1^i^	107.50 (2)
F2^ii^—Cs1—F3^i^	164.90 (3)	F2^iii^—Cs1—F1^i^	61.838 (19)
F2^i^—Cs1—F3	164.90 (3)	F2^iv^—Cs1—F1^i^	61.838 (19)
F2^ii^—Cs1—F3	105.79 (3)	F2^vi^—Cs1—F1^i^	107.50 (2)
F3^i^—Cs1—F3	59.11 (4)	F1^vii^—Cs1—F1^i^	154.77 (5)
F2^i^—Cs1—F1^iii^	69.423 (18)	F3—Br1—F3^vii^	180.0
F2^ii^—Cs1—F1^iii^	69.423 (18)	F3—Br1—F1^vii^	90.0
F3^i^—Cs1—F1^iii^	115.46 (2)	F3^vii^—Br1—F1^vii^	90.0
F3—Cs1—F1^iii^	115.46 (2)	F3—Br1—F1	90.0
F2^i^—Cs1—F1^iv^	69.423 (18)	F3^vii^—Br1—F1	90.0
F2^ii^—Cs1—F1^iv^	69.423 (18)	F1^vii^—Br1—F1	180.0
F3^i^—Cs1—F1^iv^	115.46 (2)	F2^viii^—Br2—F2^ix^	87.96 (7)
F3—Cs1—F1^iv^	115.46 (2)	F2^viii^—Br2—F2^x^	92.04 (7)
F1^iii^—Cs1—F1^iv^	120.78 (5)	F2^ix^—Br2—F2^x^	180.00 (6)
F2^i^—Cs1—F2^v^	123.869 (16)	F2^viii^—Br2—F2	180.0
F2^ii^—Cs1—F2^v^	90.05 (2)	F2^ix^—Br2—F2	92.04 (7)
F3^i^—Cs1—F2^v^	81.61 (2)	F2^x^—Br2—F2	87.96 (7)
F3—Cs1—F2^v^	57.553 (17)	F2^viii^—Br2—Cs1^xi^	120.01 (2)
F1^iii^—Cs1—F2^v^	156.305 (19)	F2^ix^—Br2—Cs1^xi^	120.005 (19)
F1^iv^—Cs1—F2^v^	58.13 (3)	F2^x^—Br2—Cs1^xi^	59.995 (19)
F2^i^—Cs1—F2^iii^	90.05 (2)	F2—Br2—Cs1^xi^	59.99 (2)
F2^ii^—Cs1—F2^iii^	123.868 (16)	F2^viii^—Br2—Cs1^xii^	59.99 (2)
F3^i^—Cs1—F2^iii^	57.553 (17)	F2^ix^—Br2—Cs1^xii^	59.995 (19)
F3—Cs1—F2^iii^	81.61 (2)	F2^x^—Br2—Cs1^xii^	120.005 (19)
F1^iii^—Cs1—F2^iii^	58.13 (3)	F2—Br2—Cs1^xii^	120.01 (2)
F1^iv^—Cs1—F2^iii^	156.305 (19)	Cs1^xi^—Br2—Cs1^xii^	180.0
F2^v^—Cs1—F2^iii^	133.81 (3)	F2^viii^—Br2—Cs1^xiii^	120.01 (2)
F2^i^—Cs1—F2^iv^	90.05 (2)	F2^ix^—Br2—Cs1^xiii^	120.005 (19)
F2^ii^—Cs1—F2^iv^	123.868 (17)	F2^x^—Br2—Cs1^xiii^	59.995 (19)
F3^i^—Cs1—F2^iv^	57.553 (17)	F2—Br2—Cs1^xiii^	59.99 (2)
F3—Cs1—F2^iv^	81.61 (2)	Cs1^xi^—Br2—Cs1^xiii^	91.951 (5)
F1^iii^—Cs1—F2^iv^	156.305 (19)	Cs1^xii^—Br2—Cs1^xiii^	88.049 (5)
F1^iv^—Cs1—F2^iv^	58.13 (3)	F2^viii^—Br2—Cs1^xiv^	59.99 (2)
F2^v^—Cs1—F2^iv^	46.64 (4)	F2^ix^—Br2—Cs1^xiv^	59.995 (19)
F2^iii^—Cs1—F2^iv^	112.26 (3)	F2^x^—Br2—Cs1^xiv^	120.005 (19)
F2^i^—Cs1—F2^vi^	123.868 (17)	F2—Br2—Cs1^xiv^	120.01 (2)
F2^ii^—Cs1—F2^vi^	90.05 (2)	Cs1^xi^—Br2—Cs1^xiv^	88.049 (5)
F3^i^—Cs1—F2^vi^	81.61 (2)	Cs1^xii^—Br2—Cs1^xiv^	91.951 (5)
F3—Cs1—F2^vi^	57.553 (17)	Cs1^xiii^—Br2—Cs1^xiv^	180.0
F1^iii^—Cs1—F2^vi^	58.13 (3)	Br1—F1—Cs1^xiii^	119.61 (2)
F1^iv^—Cs1—F2^vi^	156.305 (19)	Br1—F1—Cs1^xi^	119.61 (2)
F2^v^—Cs1—F2^vi^	112.26 (3)	Cs1^xiii^—F1—Cs1^xi^	120.78 (5)
F2^iii^—Cs1—F2^vi^	46.64 (4)	Br1—F1—Cs1^vii^	102.61 (2)
F2^iv^—Cs1—F2^vi^	133.81 (3)	Cs1^xiii^—F1—Cs1^vii^	83.807 (16)
F2^i^—Cs1—F1^vii^	147.27 (3)	Cs1^xi^—F1—Cs1^vii^	83.807 (16)
F2^ii^—Cs1—F1^vii^	57.95 (3)	Br1—F1—Cs1^i^	102.61 (2)
F3^i^—Cs1—F1^vii^	106.94 (3)	Cs1^xiii^—F1—Cs1^i^	83.807 (16)
F3—Cs1—F1^vii^	47.83 (3)	Cs1^xi^—F1—Cs1^i^	83.807 (16)
F1^iii^—Cs1—F1^vii^	96.194 (16)	Cs1^vii^—F1—Cs1^i^	154.78 (5)
F1^iv^—Cs1—F1^vii^	96.194 (16)	Br2—F2—Cs1^i^	179.32 (6)
F2^v^—Cs1—F1^vii^	61.838 (19)	Br2—F2—Cs1^xiii^	90.42 (3)
F2^iii^—Cs1—F1^vii^	107.50 (2)	Cs1^i^—F2—Cs1^xiii^	89.95 (2)
F2^iv^—Cs1—F1^vii^	107.50 (2)	Br2—F2—Cs1^xi^	90.42 (3)
F2^vi^—Cs1—F1^vii^	61.838 (19)	Cs1^i^—F2—Cs1^xi^	89.95 (2)
F2^i^—Cs1—F1^i^	57.95 (3)	Cs1^xiii^—F2—Cs1^xi^	112.26 (3)
F2^ii^—Cs1—F1^i^	147.27 (3)	Br1—F3—Cs1^i^	119.56 (2)
F3^i^—Cs1—F1^i^	47.83 (3)	Br1—F3—Cs1	119.56 (2)
F3—Cs1—F1^i^	106.94 (3)	Cs1^i^—F3—Cs1	120.89 (4)
F1^iii^—Cs1—F1^i^	96.194 (16)		

Symmetry codes: (i) −*x*+1, −*y*+1, −*z*+1; (ii) *x*, *y*, −*z*+1; (iii) *x*+1/2, *y*+1/2, *z*+1/2; (iv) *x*−1/2, *y*+1/2, *z*+1/2; (v) −*x*+1/2, −*y*+1/2, *z*+1/2; (vi) −*x*+3/2, −*y*+1/2, *z*+1/2; (vii) −*x*+1, −*y*, −*z*+1; (viii) −*x*+1, −*y*, −*z*; (ix) *x*, *y*, −*z*; (x) −*x*+1, −*y*, *z*; (xi) *x*+1/2, *y*−1/2, *z*−1/2; (xii) −*x*+1/2, −*y*+1/2, −*z*+1/2; (xiii) *x*−1/2, *y*−1/2, *z*−1/2; (xiv) −*x*+3/2, −*y*+1/2, −*z*+1/2.
